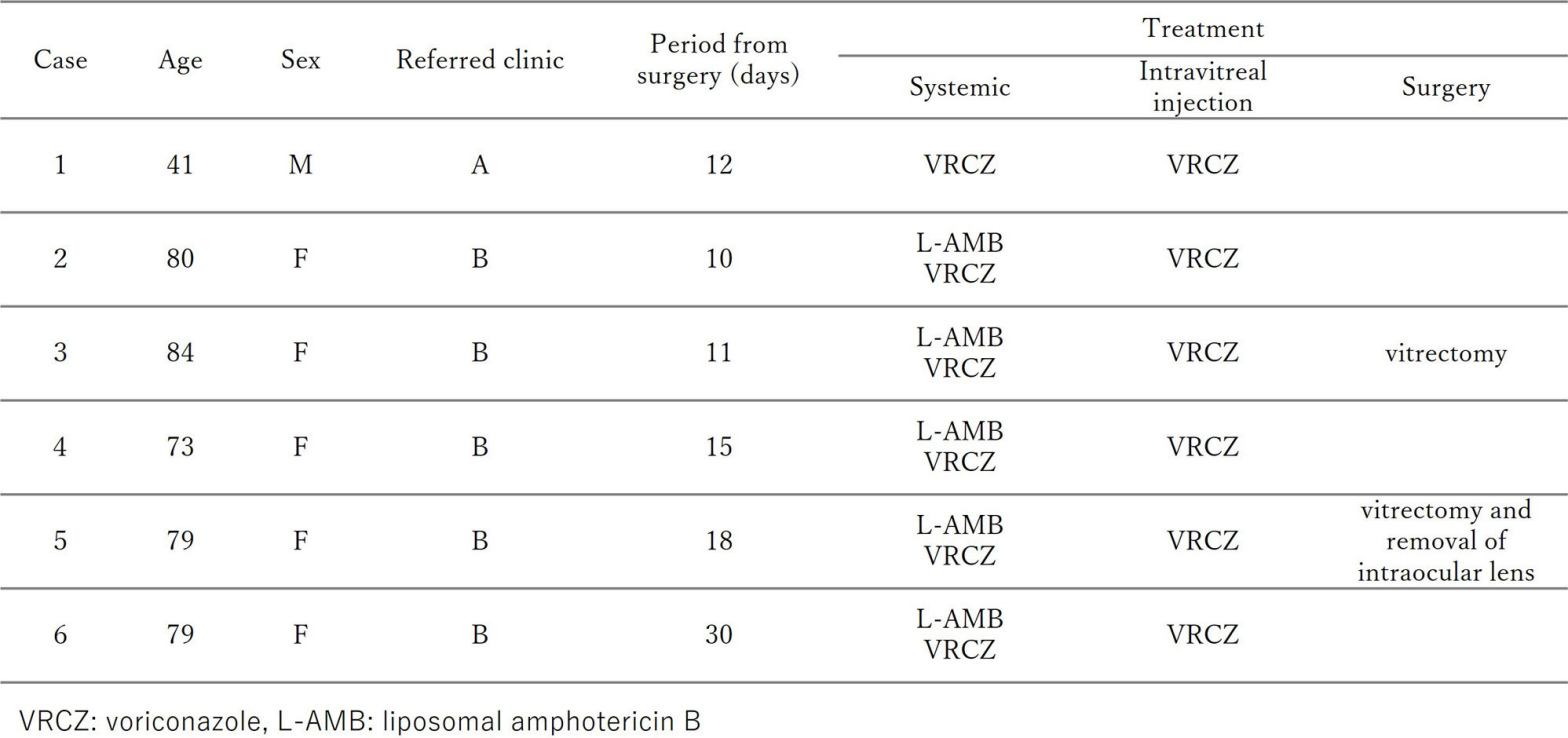# 333 Institutional and Behavioral Determinants of Sharps Injury Reporting and Protective Equipment Use in Nursing Professionals in Croatia

**DOI:** 10.1017/ash.2026.10677

**Published:** 2026-06-23

**Authors:** Teppei Shimasaki

**Affiliations:** 1 Kyorin University

## Abstract

**Background:** Trypan blue is commonly used during mature cataract surgery to improve visualization of the anterior capsule. Although FDA-approved since 2004, it remains unapproved in Japan. Many clinics perform sterile filtration prior to use; however, no standardized sterilization protocol exists. **Methods:** This retrospective case series included all patients diagnosed with Sarocladium kiliense endophthalmitis after cataract surgery at Kyorin University Hospital between 2015 and 2025. **Results:** In 2025, six microbiologically confirmed cases of S. kiliense endophthalmitis were identified; none occurred before 2024. One case originated from Clinic A and five from Clinic B. All cataract surgeries used trypan blue, which had been sterile-filtered at each institution. Discussion: Endophthalmitis is a rare postoperative complication of cataract surgery, and S. kiliense is an uncommon pathogen. We were informed that similar cases occurred sporadically in 2025 from a reference laboratory center. One institution confirmed contamination of the trypan blue product (unpublished data). The same organism was isolated from dye obtained from Clinic B. All affected clinics used trypan blue from the same manufacturer. Off-label product use may pose significant safety concerns due to limited data. While other off-label dyes (e.g., Lugol’s solution, India ink) are widely used in Japan, they are applied superficially. We hypothesize that contaminated trypan blue migrated into the vitreous cavity via lens zonules, causing the outbreak. Physicians bear responsibility for off-label use and should employ unapproved products only when benefits clearly outweigh risks.

**Figure:**